# Tissue-resident memory T cells in atopic dermatitis: mechanisms of residual inflammation, relapse, and therapeutic persistence

**DOI:** 10.3389/fimmu.2026.1849265

**Published:** 2026-07-20

**Authors:** Natalia Zdanowska, Natalia Machoń, Alicja Frączek, Marta Kasprowicz-Furmańczyk, Joanna Czerwińska, Agnieszka Owczarczyk-Saczonek

**Affiliations:** 1 Department of Dermatology, Sexually Transmitted Diseases and Clinical Immunology, University of Warmia and Mazury in Olsztyn, Olsztyn, Poland; 2 Doctoral School of the University of Warmia and Mazury in Olsztyn, Olsztyn, Poland

**Keywords:** atopic dermatitis, chronic inflammation, disease memory, relapse, tissue-resident memory T cells

## Abstract

**Background:**

Atopic dermatitis (AD) is a chronic, relapsing inflammatory skin disease in which disease flares frequently recur at previously affected anatomical sites. This distinctive clinical pattern suggests the presence of residual inflammation and long-lasting local immune memory that persists beyond apparent clinical remission.

**Objectives:**

To synthesize current evidence on the role of tissue-resident memory T (TRM) cells in the pathogenesis, chronicity, and relapse of AD, and to discuss how TRM biology may explain disease recurrence after treatment withdrawal and inform future therapeutic strategies.

**Methods:**

Narrative review of experimental, translational, and clinical studies addressing TRM differentiation, persistence, metabolic and epigenetic programming, clonal stability, microenvironmental crosstalk, and treatment-related modulation in AD and related chronic dermatoses.

**Results:**

TRM are long-lived, non-circulating T cells retained in the skin through adhesion molecules, survival cytokines, metabolic adaptation, and stable epigenetic programs. In AD, both CD4+ and CD8+ TRM persist in lesional and clinically resolved skin and remain transcriptionally poised for rapid reactivation. Clinical studies consistently demonstrate disease relapse after discontinuation of biologics and JAK inhibitors, supporting the concept that current therapies suppress inflammatory pathways without eliminating pathogenic tissue memory. Emerging data suggest that selected interventions, including modulation of costimulatory pathways and survival signals, may partially influence immune memory and prolong disease control in subsets of patients.

**Conclusion:**

TRM constitute a central cellular substrate of residual disease memory in AD and provide a mechanistic explanation for site-specific relapse and treatment resistance. Therapeutic strategies that selectively modulate pathogenic TRM and their supporting microenvironment, while preserving protective barrier immunity, may be required to achieve durable remission.

## 1 Introduction

Atopic dermatitis (AD) is one of the most prevalent chronic inflammatory skin diseases, affecting up to 20% of children and approximately 10% of adults worldwide, with a steadily increasing incidence in industrialized regions (1, 2). Beyond its cutaneous manifestations, AD is associated with a substantial psychosocial and socioeconomic burden, including impaired quality of life, sleep disturbance, reduced work productivity, and increased prevalence of anxiety and depression (1). Over the past decade, the introduction of targeted biologics blocking type 2 cytokine signaling and small-molecule Janus kinase (JAK) inhibitors has significantly improved disease control for many patients. Nevertheless, relapse after treatment discontinuation remains common, underscoring the limitations of current therapies in inducing durable remission (2, 3).

A striking clinical feature of AD is the recurrence of lesions at identical anatomical sites, even after apparent clinical resolution. While several generalized mechanisms contribute to overall disease chronicity, such as persistent IgE-mediated sensitization, ongoing systemic skin barrier dysfunction, or neuro-immune pathways (4). These factors alone fail to adequately explain this strict spatial precision. Instead, the phenomenon of site-specific recurrence points directly to a localized form of residual disease memory (4).

This phenomenon has prompted growing interest in the concept of residual disease memory, increasingly attributed to tissue-resident memory T (TRM) cells. TRM are long-lived, non-circulating lymphocytes embedded within peripheral tissues such as the skin, where they maintain local immune programs and enable rapid recall responses (3, 5, 6). While TRM have been extensively characterized in psoriasis and other immune-mediated dermatoses, their contribution to AD pathogenesis and relapse has only recently begun to be elucidated. Accumulating evidence indicates that persistent TRM populations in both lesional and non-lesional skin may represent the cellular substrate underlying chronicity and site-specific relapse in AD. Given the distinct immune polarization, such as stronger Th17 skewing, and differential anatomical distribution of lesions in pediatric populations, the scope of this review is strictly confined to adult patients with AD (4).

### 1.1 Disease burden and quality of life in atopic dermatitis

Atopic dermatitis represents a major cause of non-fatal skin-related disability worldwide. According to the Global Burden of Disease (GBD) Study 2021, approximately 129 million individuals were affected by AD in 2021, with projections estimating an increase to 148 million cases by 2050, highlighting the expanding public health burden of this disease (7). Earlier GBD analyses ranked AD as the leading skin disease in terms of disability-adjusted life years and among the most burdensome non-fatal conditions overall (8).

Beyond prevalence, AD profoundly affects daily functioning and societal productivity. Large cross-sectional surveys from Europe and the United States demonstrated a strong association between disease severity and work productivity loss, with progressively greater weekly reductions in productive time from mild to severe disease (9). Data from the multicountry ESSENTIAL AD study further confirmed substantial clinical, psychosocial, and economic burden irrespective of treatment modality, with particularly high psychosocial impact among patients requiring systemic therapy (10).

Quality-of-life impairment in AD is largely driven by chronic pruritus and sleep disturbance. A recent meta-analysis reported sleep disorders in up to 43.4% of patients with AD, with prevalence increasing in parallel with disease severity (11). Importantly, the consequences extend beyond affected individuals. Long-term cohort data demonstrate impaired sleep quality and daytime functioning among caregivers, particularly mothers of children with AD (12). Chronic sleep deprivation resulting from persistent itch contributes to physical exhaustion, emotional distress, impaired concentration, and reduced productivity, amplifying the overall disease burden (13).

Psychiatric comorbidity further compounds the impact of AD. A systematic review and meta-analysis identified significantly increased risks of depression, anxiety, and suicidal ideation among adults with AD, while pediatric populations also showed positive associations between AD and depressive symptoms (14). Large population-based studies revealed a dose–response relationship between AD severity and incident psychiatric disease, emphasizing the importance of sustained disease control and relapse prevention as clinically meaningful therapeutic goals (15).

### 1.2 Relapse after treatment withdrawal

Despite substantial therapeutic advances, relapse after treatment discontinuation remains a hallmark of AD management, reflecting incomplete eradication of pathogenic immune memory. While current systemic therapies effectively suppress active inflammation, clinical and transcriptomic evidence suggests they largely fail to clear the underlying reservoir of tissue-resident memory T (TRM) cells, which act as a local “alarm system” poised to re-initiate the disease (16–20).

Dupilumab, a monoclonal antibody targeting IL-4 receptor α and inhibiting IL-4 and IL-13 signaling, has demonstrated robust efficacy in moderate-to-severe AD (21). However, the SOLO-CONTINUE study showed that patients switched from maintenance dupilumab to placebo gradually lost disease control, with early re-emergence of pruritus and relapse in most patients during follow-up EASI-75 was maintained by 30.4% on placebo and by 71.6% with continued dupilumab. The time to first loss of IGA 0/1 was a mean of 57 days in the placebo group compared to 114 days with continued dupilumab (21). Real-world cohorts indicate that sustained remission after discontinuation is uncommon, although Japanese studies have reported prolonged remission in a minority of patients treated with topical therapy alone after cessation (22, 23). After stopping dupilumab 49% patients worsened and required retreatment, with mean time to exacerbation about 16.2 weeks (24). Recent real-world data confirm these timelines, showing a median time to relapse of up to 29 weeks depending on individual risk factors (25).

Crucially, this gradual relapse aligns with transcriptomic studies demonstrating that clinically resolved AD skin is still characterized by the persistence of TRM cells (18–20).

JAK inhibitors provide rapid suppression of inflammatory signaling and pruritus, yet withdrawal studies consistently demonstrate early disease recurrence. In the REGIMEN study, discontinuation or dose reduction of abrocitinib increased flare probability, with a median time to flare of about 28 days (17). Similar findings were reported for upadacitinib, with pruritus worsening within approximately 5 days and loss of skin response within approximately 4 weeks after discontinuation (24–26).

For tralokinumab, long-term extension studies demonstrated superior maintenance of response with continued therapy compared with placebo Among responders reassigned to placebo, tralokinumab maintained an IGA 0/1 score in 34.0% and an EASI-75 score in 26.4% at week 52 (27). Similar patterns have been observed with lebrikizumab, with maintenance of IGA 0/1 in 47.9% and EASI-75 in 66.4% (28).

Collectively, the withdrawal kinetics of these targeted therapies, ranging from days for JAK inhibitors to weeks or months for biologics, reflect the temporal dynamics of residual TRM reactivation rather than purely pharmacokinetic wash-out (16, 17, 29).

Amlitelimab, targeting the OX40–OX40L axis, represents a distinct approach aimed at modulating pathogenic T-cell memory. OX40 expression on skin-homing memory T cells has been demonstrated in AD. In the STREAM-AD phase 2b trial, 57.0% of patients who discontinued amlitelimab retained an IGA 0/1 score, whereas 61.6% maintained EASI-75 (30–33).

In contrast, nemolizumab, targeting IL-31 receptor A, primarily modulates neuroimmune pathways and provides rapid itch control. Memory T cells are recognized as a major source of IL-31 in AD (34–38).

## 2 Biology of tissue-resident memory T cells

Human skin serves as a primary reservoir for TRM cells, harboring distinct epidermal and dermal subsets that exhibit characteristic phenotypic and functional characteristics. Epidermal TRM are predominantly CD8+ and characterized by high expression of CD103, which facilitates their tight anchoring to keratinocytes within the stratified epithelium. These cells are functionally poised for rapid cytotoxic responses and the production of IFN-γ. In contrast, dermal TRM are more frequently CD4+ and typically reside in the papillary dermis. Unlike their epidermal counterparts, dermal TRM often lack CD103 expression, as the dermal environment lacks the high E-cadherin levels found in the epidermis. The immunological nature of atopic dermatitis, however, provides important differences to these conventional patterns. Although generally considered an epidermal CD8+ marker, pathogenic CD4+ CD103+ TRM populations have been observed in the skin of AD patients. These cells appear to bridge the anatomical compartments and are potent producers of IL-4, IL-13 and IL-22, driving both barrier dysfunction and chronic pruritus. Functionally, these diverse TRM subsets are transcriptionally poised for immediate reactivation, enabling them to shape the local inflammatory milieu during secondary immune responses (39). Interestingly, the emerging concept of “ex-TRM”, which are circulating cells with a TRM-like phenotype, challenges the traditional view of strict tissue confinement. These cells suggest a level of plasticity where resident populations may exit the skin under specific inflammatory conditions while retaining their homing signatures (40, 41). This phenotypic and spatial heterogeneity underscores the complexity of TRM biology in AD, where the interplay between epidermal CD8+ cells and specialized dermal CD4+ populations creates a self-sustaining reservoir of disease memory (**Supplementary Table 1**).

### 2.1 TRM in skin immunity and chronic dermatoses

Human skin contains billions of T lymphocytes, among which TRM form a dominant non-circulating population that provides rapid, site-specific immune protection (3, 42). In protective contexts, CD8+ TRM specific for viral antigens persist within the epidermis and mediate immediate recall responses against pathogens such as herpes simplex virus, while IL-17–producing CD4+ TRM contribute to antifungal defense against Candida albicans Tumor-infiltrating TRM have also been associated with durable immune surveillance in melanoma (42, 43).

Conversely, the same properties that confer protection enable TRM to act as pathogenic amplifiers in chronic inflammatory dermatoses. A defining feature of TRM biology is their ability to establish site-specific disease memory, resulting in recurrence of lesions at identical anatomical locations despite clinical resolution (5, 44). In psoriasis, epidermal CD8+ CD69+ CD103+ TRM persist in resolved lesions and retain the capacity to produce IL-17 and IL-22. In vitiligo, melanocyte-reactive CD8+ TRM mediate depigmentation via IFN-γ– and granzyme B–dependent pathways. Similar mechanisms operate in alopecia areata (45–52), fixed drug eruption, and cutaneous T-cell lymphoma (17, 53–56).

**Figure 1:**
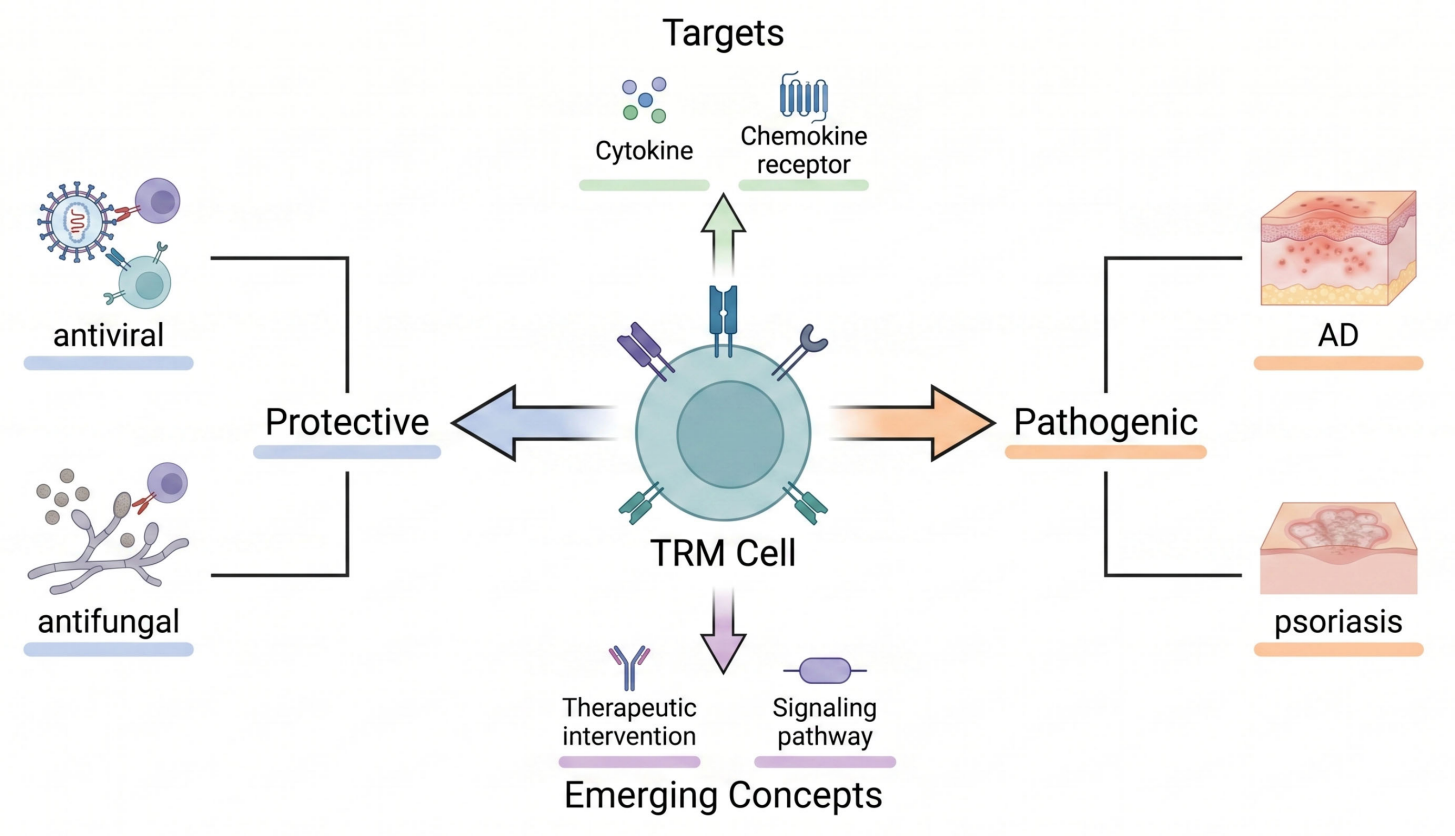
**Protective versus pathogenic roles of TRM in skin immunity** (2, 4–7, 11, 13, 14). Created in BioRender. Zdanowska, N. (2026) https://BioRender.com/phw70am This schematic illustrates the dual role of TRM in skin immunity. On the protective side, CD8+ TRM provide rapid defense against viral infections such as herpes simplex virus, IL-17–producing CD4+ TRM mediate antifungal protection against *Candida albicans*, and tumor-infiltrating TRM contribute to durable melanoma surveillance. On the pathogenic side, both CD4+ and CD8+ TRM amplify chronic inflammation in autoimmune and allergic dermatoses. In atopic dermatitis, Th2-skewed CD4+ TRM produce IL-4, IL-13, and IL-22, driving barrier dysfunction and relapse. In psoriasis, CD8+ TRM secrete IL-17 and IL-22, maintaining site-specific memory and recurrence. In vitiligo, autoreactive TRM release IFN-γ, granzyme B, and perforin, contributing to melanocyte destruction. TRM are also implicated in alopecia areata, fixed drug eruption, and cutaneous T-cell lymphoma, highlighting their potential pathogenic and oncogenic roles. Together, the figure underscores the dichotomy of TRM biology as both indispensable guardians of barrier immunity and central drivers of recurrent skin inflammation.

In atopic dermatitis, both CD4+ and CD8+ TRM persist in lesional and clinically resolved skin, where they remain transcriptionally poised and capable of rapid cytokine production (2, 5). T-cell receptor sequencing demonstrates shared dominant clonotypes between lesional and non-lesional skin that persist after successful therapy, supporting the concept of widespread residual immune memory (18–20) (**Figure 1**).

Given their dual role in both protective immunity and chronic inflammation, TRM have been characterized across multiple skin conditions, ranging from infections and tumors to autoimmune and allergic dermatoses (**Supplementary Table 2**).

### 2.2 Mechanisms of TRM persistence in atopic dermatitis

**Figure 2:**
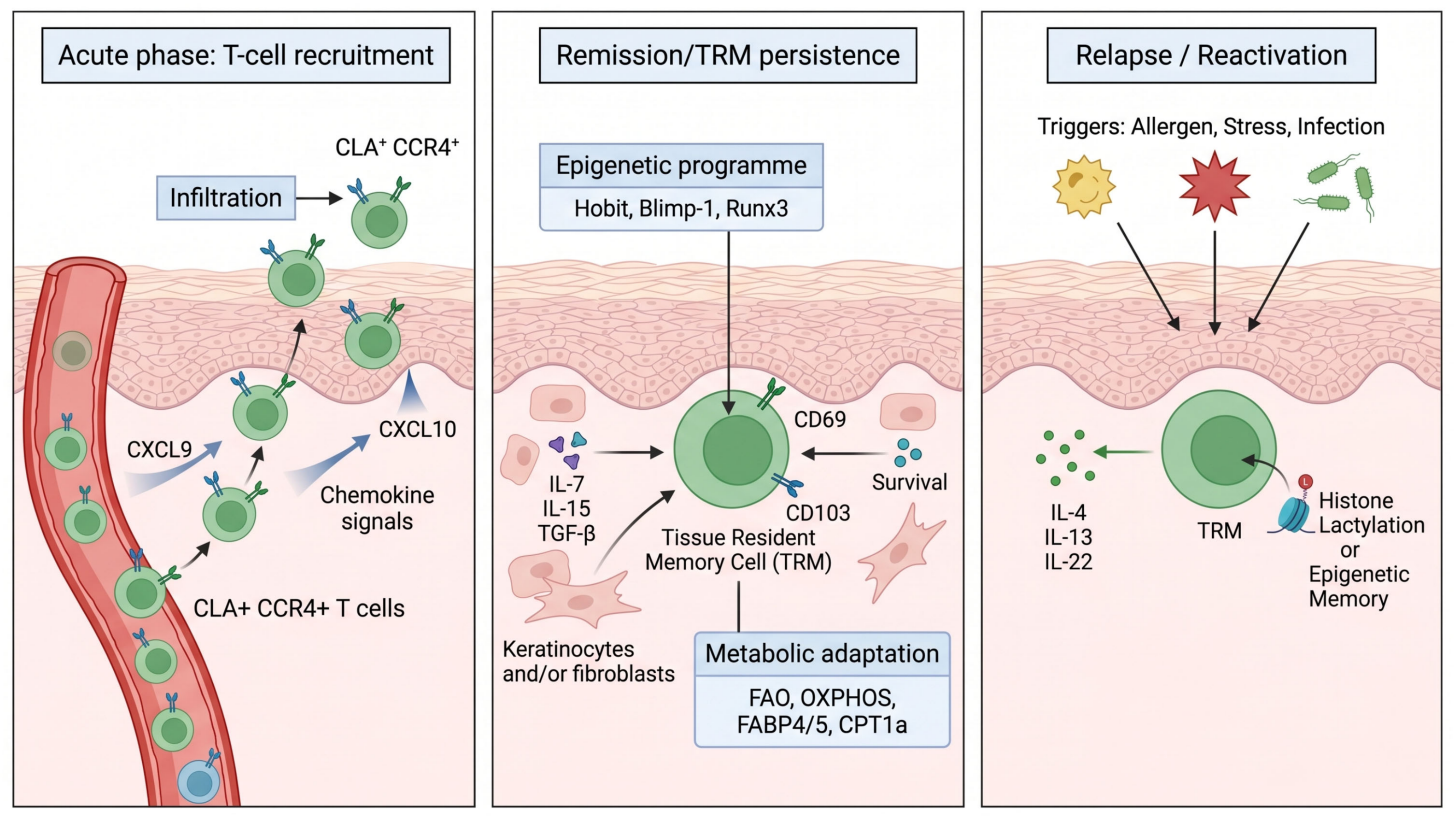
Mechanisms of TRM persistence in atopic dermatitis. Created in BioRender. Zdanowska, N. (2026) https://BioRender.com/ravab87. This schematic summarizes the mechanisms underlying TRM persistence in atopic dermatitis. Anchoring signals secure TRM within the skin: CD69 antagonizes S1PR1 to block egress, CD103 binds keratinocyte-derived E-cadherin, and CD49a interacts with collagen IV in the basement membrane. Survival cues are provided by cytokines IL-7, IL-15, and TGF-β, while epithelial alarmins (IL-25, IL-33, TSLP) further amplify persistence. TRM exhibit metabolic adaptation by relying on fatty acid uptake and mitochondrial oxidative phosphorylation (OXPHOS) supported by FABP4/5 and CPT1a, ensuring long-term survival. Epigenetic programming, including the action of Hobit, Blimp-1, and Runx3, locks TRM into a poised effector state. Crosstalk with keratinocytes, dendritic cells, mast cells, and ILC2 amplifies pathogenic function, perpetuating chronic inflammation and site-specific relapse in AD.

The persistence of TRM cells in AD can be classified into intracellular programs and external interactions with the skin microenvironment (1–3, 5). Intracellular programs include key factors such as Hobit, Blimp-1, and Runx3, which maintain the cells in a “ready” effector state. These programs facilitate rapid reactivation upon re-exposure to an allergen, while simultaneously downregulating genes required for exiting the tissue (3, 5). Physical retention in epithelial niches is further secured by a specific surface marker such as CD69, which antagonizes sphingosine-1-phosphate receptor 1 and prevents tissue egress, CD103 (αE integrin), which anchors cells to epithelial E-cadherin, and CD49a (VLA-1), which mediates adhesion to collagen IV in the basement membrane (3, 5, 16). Additionally, TRM cells exhibit remarkable metabolic efficiency, shifting their energy consumption toward fatty acid uptake and mitochondrial oxidative phosphorylation. The persistence of these internal programs is confirmed by T cell receptor (TCR) sequencing, which reveals that identical clonotypes are maintained in resolved AD lesions for months, constituting a stable “molecular trace” of disease memory (1–3). The major mechanisms responsible for TRM persistence and reactivation in AD are summarized in **Figure 2**.

Beyond intrinsic programming, the persistence of TRM cells is further reinforced by a continuous dialogue with surrounding non-immune cells. Keratinocytes, fibroblasts, and hair follicle cells function as a primary “maintenance support” system by secreting IL-7 and IL-15, both of which are indispensable for TRM homeostatic proliferation and long-term viability (2, 5, 57). Keratinocytes further stabilize this niche by producing chemokines such as CCL20 and CCL27, effectively anchoring resident cells within the epidermal and dermal compartments (2, 58). Furthermore, the local production of TGF-β within the microenvironment is essential to induce and maintain the expression of the retention marker CD103, effectively tethering the cells to the epithelium. Additionally, chronic barrier disruption observed in the course of atopic dermatitis stimulates the release of epithelial interleukins, including IL-25, IL-33, and Thymic Stromal Lymphopoietin (TSLP). These signals create a self-perpetuating inflammatory loop that not only strengthens the existing TRM population but also promotes the differentiation of newly recruited effector cells into a resident memory phenotype by inducing keratinocytes to produce eotaxin-3 (59). Additionally, the secretion of IL-31 serves as a critical link between immune activation, barrier impairment, and the sensation of pruritus (37). This system is further modulated by microbial and neuronal inputs. Components of *Staphylococcus aureus*, frequently found in AD lesions, induce TSLP and directly trigger mast cell activation, exacerbating the inflammatory state (60, 61). Simultaneously, neuropeptides regulate ILC2 activity, linking stress and itch perception with immune memory (62, 63). Finally, the microenvironment dictates tissue architecture through specific chemokine gradients that facilitate the initial homing of effector T cells; once these cells infiltrate the tissue, local crosstalk instructs them to adopt residency markers, integrating them permanently into the existing cutaneous immune network (2, 5).

## 3 Therapeutic modulation of inflammation and disease memory in atopic dermatitis

Current therapies effectively suppress inflammatory signaling but rarely eradicate pathogenic TRM reservoirs. Treatment with biologics and JAK inhibitors reduce clinical inflammation and improve molecular disease signatures. However, the relapse after treatment withdrawal is common. This pattern supports the concept of persistent residual inflammatory activity in the skin. Rather, it is believed that TRM cells likely participate in a broader relapse-prone tissue ecosystem that involves impaired barrier function, epithelial-derived alarmins, IgE-mediated sensitization, mast cells, microbial signals and neuroimmune mechanisms (18–20, 64).

Conventional skin-directed therapies exert partial effects. Topical corticosteroids and vitamin D analogues reduce inflammatory infiltrates and TRM-associated markers, particularly in the epidermis, but deeper resident populations often persist (65, 66). Narrow-band UVB phototherapy improves clinical and molecular features of AD without selectively eliminating TRM (67). Because most withdrawal studies were not focused on direct measuring of TRMs, biopsy-based and spatially resolved longitudinal studies are essential to determine whether relapse reflects persistence of TRMs, reactivation of recruited T cells, barrier abnormalities or other residual immune mechanisms (18–20, 64, 68, 69).

Emerging strategies aim to directly modulate TRM persistence. Targeting adhesion pathways, survival cytokines (notably IL-15 and TGF-β), metabolic dependencies, and epigenetic programs represents promising avenues for selectively influencing disease memory (70–74). Among those systemic therapies, targeting the OX40-OX40L pathway appears to be the most relevant taking into consideration disease memory, because this pathway includes T-cell activation, survival and the persistence of pathogenic T-cell responses. Amlitelimab, a monoclonal antibody directed against OX40L has shown maintaining clinical responses even after discontinuation of treatment (30, 32, 33). These studies demonstrate that modulation of costimulatory signaling may have a more durable effect on pathogenic T-cell programs than inhibition of downstream effector cytokines alone. However, the available evidence doesn’t demonstrate selective depletion or permanent reprogramming of pathogenic TRM cells. Therefore, amlitelimab is best described as a clinically advanced strategy with potential memory-modulating effects, rather than a TRM eradicating therapy (31).

Another biologically plausible target for therapies aimed at TRM cells are survival cytokines. IL-15 is particularly interesting, because it plays an important role in supporting the maintenance and long-term survival of TRM cells within peripheral tissues. Studies on vitiligo show that blocking the IL-15/CD122 axis reduces the autoreactive TRM activity, suppress IFN- γ-associated tissue memory and contributes to more durable disease control (71, 75, 76). These findings show that interfering with TRM survival signals can potentially modify tissue memory. Therefore IL-15 targeting in AD should be considered as a hypothesis rather than an established therapeutic strategy.

Adhesion and retention pathways may also represent potential therapeutic targets. CD69 promotes tissue retention by antagonizing S1PR1 and limiting tissue egress. CD103 supports epithelial anchoring through binding to E-cadherin. CD49a facilitates interaction with collagen type IV in the basement membrane (3, 5, 16, 70). Theoretically those interferences could weaken pathogenic tissue residency. However, it worth mentioning that those pathways are not disease specific. They play an important role in protective immune surveillance, like antimicrobial or antitumor responses at barrier sites. Accordingly, broad inhibiting of TRM retention could compromise beneficial barrier immunity.

Therefore, in AD, modulating the tissue niches that support pathogenic TRM persistence could be a better strategy that blocking retention pathways. These niches include keratinocyte-derived IL-7 and IL-15, chemokines, epithelial alarmins and stromal signals (57, 58, 75, 77, 78).

Another interesting area is metabolic targeting. The survival of TRM cells depends in some part on exogenous lipid uptake, fatty-acid-binding proteins (like FABP4 and FABP5), CPT1a dependent fatty acid oxidation and mitochondrial oxidative phosphorylation. These metabolic pathways may help TRM cells persist in clinically resolved skin, even when inflammatory cytokine signaling is suppressed. Targeting that area could theoretically reduce long-term survival. This approach however remains experimental, and more AD-specific research need to be done (18–20, 39, 73).

Epigenetic reprogramming seems to be another relevant direction. TRM identity, persistence and rapid effector capacity are maintained by transcriptional and epigenetic mechanisms involving Runx3, Hobit, Blimp-1, chromatin accessibility, histone modifications, DNA methylation and non-coding RNAs (3, 5, 74). It leads to the conclusion that the aim would not be to eliminate all TRM cells, because they are also essential for antimicrobial and antitumor immunity, but to selectively reprogram pathogenic TRM states or weaken disease associated inflammatory memory. Therefore, interventions targeting Runx3, Hobit or bimp-1 should be taken into consideration and more research need to be performed (74).

Summing up, TRM-directed therapies in AD are more focused on niche therapy, because TRM persistence is possible due to interactions with keratinocytes, fibroblasts, dendritic cells, ILC2, nerves, the microbiota and hair follicle-derived survival signals. For this reason, modifying the microenvironment may be a better strategy than directly eliminating TRM cells (37, 57–63, 75, 77, 78).

## 4 Conclusion, perspectives, and methodological considerations

Tissue-resident memory T cells provide a unifying immunological framework linking residual inflammation, site-specific relapse, and treatment resistance in atopic dermatitis. Current treatments primarily suppress inflammatory pathways without reliably eliminating pathogenic TRM reservoirs, supporting the need for maintenance strategies in many patients.

From a therapeutic perspective, approaches targeting costimulatory pathways, survival cytokines, metabolic fitness, and epigenetic programming may enable selective modulation of pathogenic tissue memory while preserving protective barrier immunity. TRM also represent potential biomarkers for relapse risk and therapeutic responsiveness, particularly given the emerging concept of circulating TRM-like (“ex-TRM”) populations. However, translation into clinical practice requires further validation.

Several methodological challenges complicate TRM research in humans, including limited standardization of marker definitions, phenotypic plasticity of resident populations, and reliance on cross-sectional biopsy analyses. Integrative approaches combining surface phenotyping with transcriptomic, epigenetic, and clonal analyses, as well as advances in single-cell and spatial multi-omics, longitudinal sampling, and clonotype tracking, are expected to refine TRM classification and improve reproducibility.

In conclusion, TRM biology provides both an explanation for the relapsing nature of AD and a roadmap for future therapeutic innovation. Strategies aimed at selectively reprogramming pathogenic tissue memory, rather than indiscriminate immunosuppression, may ultimately enable durable remission and long-term restoration of skin homeostasis.
