# Supplementary material for: Tissue-resident memory T cells in atopic dermatitis: mechanisms of residual inflammation, relapse, and therapeutic persistence
Source: Front Immunol. 2026 Jul 20;17:1849265. doi: 10.3389/fimmu.2026.1849265 (PMC13429481; doi:10.3389/fimmu.2026.1849265)
Supplement: Supplementary file 1 [file Table1.docx]

Supplementary Material

# Supplementary Data

Supplementary Table 1. Phenotypic and functional features of skin TRM subsets. ~~[3,4,29,31,33].~~

| **Marker** | **Function** | **Dominant subsets** | **Cytokines produced** |
| --- | --- | --- | --- |
| CD69 | Prevents tissue egress via antagonism of S1PR1; marker of residency | CD4+ and CD8+ TRM | IL-4, IL-13, IL-17, IL-22, IFN-γ |
| CD103 (αE integrin) | Anchors CD8+ TRM to keratinocytes via E-cadherin; promotes epithelial retention | Mainly CD8+ TRM in epidermis | IFN-γ, TNF-α |
| CD49a (VLA-1) | Adhesion to collagen IV in basement membrane; linked to cytotoxic potential | Subsets of CD8+ TRM | IL-17, IL-22 |
| PD-1 | Immune checkpoint molecule; associated with enhanced effector capacity in AD | Subsets of CD4+ and CD8+ TRM in AD lesions | IL-4, IL-13, IL-17, IL-22 |

**Legend:**
This table summarizes the major surface markers and immune functions of TRM cells in human skin. CD69 and CD103 are canonical markers of residency, CD49a marks subsets with enhanced cytotoxic potential, and PD-1 reflects immune checkpoint modulation. Differences between CD4+ and CD8+ TRM, as well as their cytokine production profiles, highlight their heterogeneity and distinct roles in protective versus pathogenic immunity.

**Supplementary Table 2. TRM subsets in skin diseases: protective and pathogenic roles ~~[2,4,29,30,33,34,50,51].~~**

| Disease/Condition | Dominant TRM subset | Dominant inflammatory axis | Main cytokines/effectors | Role |
| --- | --- | --- | --- | --- |
| Atopic dermatitis (AD) | CD4+ Th2-skewed; CD8+ multifunctional | Type 2 / barrier - associated inflammation | IL-4, IL-13, IL-22, IL-17 | Pathogenic – residual allergic/type 2 tissue memory, relapse after therapy |
| Psoriasis | Epidermal CD8+ CD69+ CD103+ TRM and IL-17 producing T-cell subsets | IL-23 / IL-17 | IL-17A, IL-22, TNF- α | Pathogenic site-specific psoriatic memory and recurrence at identical previously affected sites |
| Vitiligo | CD8+ melanocyte-reactive TRM secreting IFN-γ, granzyme B, perforin | IFN-γ / CXCL9-CXCL10 axis | IFN-γ, granzyme B, perforin | Pathogenic – melanocyte destruction |
| Alopecia areata | CD8+ CD103+ TRM clustering around hair follicles | IFN-γ / JAK - dependent cytotoxic axis | IFN-γ, TNF-α, cytotoxic mediators | Pathogenic – hair follicle autoimmunity |
| Fixed drug eruption | CD8+ intraepidermal TRM with effector-memory features | Drug-specific cytotoxic recall response | IFN-γ, TNF-α, granzyme B | Pathogenic – localized recurrence upon drug re-exposure |
| Cutaneous T-cell lymphoma (CTCL) | Transformed clonal TRM-like clones (CD4+ or CD8+) | Variable, disease-stage dependent | Variable (malignant clones) | Pathogenic/oncogenic tissue-resident clonal expansion |
| HSV infection | CD8+ epidermal TRM | Antiviral type 1 immunity | IFN-γ, cytotoxic mediators | Protective – antiviral defense |
| Candida albicans infection | CD4+ IL-17-producing dermal TRM | Antifungal IL-17 axis | IL-17 | Protective – antifungal defense |
| Melanoma surveillance | CD8+ cytotoxic TRM | Antitumor cytotoxic immunity | IFN-γ, TNF-α, cytotoxic mediators | Protective – long-term tumor surveillance |

**Legend:**
This table summarizes the contribution of TRM subsets in major skin diseases and conditions. While CD8+ TRM mediate protective immunity against viral and fungal pathogens and provide tumor surveillance in melanoma, both CD4+ and CD8+ TRM contribute to the recurrence and chronicity of inflammatory dermatoses such as AD, psoriasis, vitiligo, alopecia areata, fixed drug eruption, and CTCL. The dichotomy of TRM functions underscores their role as both indispensable defenders of barrier immunity and central drivers of chronic disease.
